# Rehabilitation of Acquired Maxillary Defect With Patient-Specific Implants Post-mucormycosis Resection: A Case Report

**DOI:** 10.7759/cureus.70784

**Published:** 2024-10-03

**Authors:** Deepesh Saxena, Avi Pahwa, Ayush Kumar

**Affiliations:** 1 Department of Prosthodontics and Crown and Bridge, Subharti Dental College and Hospital, Swami Vivekanand Subharti University, Meerut, IND; 2 Department of Prosthodontics and Implantology, Clove Dental Clinic, Bangalore, IND

**Keywords:** defect, maxillofacial prosthesis implantation, palatal obturator, patient-specific implant, rhino-orbital mucormycosis

## Abstract

The outbreak of COVID-19 swept massive masses causing rhinocerebral mucormycosis, a fatal mycotic infection, and high mortality among humans. ​It affects individuals with weakened immune systems, resulting in severe ulcers on the palate or perforation of the palate, accompanied by blackish necrotic tissue and exposure of underlying bone.​ Some of the key symptoms include rhinorrhea, facial swelling, pain in the orofacial region, varying degrees of fever, headaches, blurred vision due to proptosis, and involvement of the contents of the orbit. The impairment is enormous and thus needs a surgical resection of the palate and associated structures, leaving the patient with both compromised function and psychological impairment. This case report presents a new digital technique for the rehabilitation of acquired maxillary defects with patient-specific implants.

## Introduction

Maxillary rehabilitation following resection due to mucormycosis presents a significant challenge in the oral and maxillofacial discipline. The global impact of mucormycosis surged during the COVID-19 pandemic, with many immunocompromised patients developing this life-threatening infection. According to epidemiological data, countries like India reported over 47,000 cases of mucormycosis post-COVID-19, with mortality rates ranging between 30% and 50% [1]. The necessity for maxillary resections after mucormycosis highlights the relevance of advanced rehabilitation techniques to restore both function and aesthetics.

Traditional methods, such as augmentation with free bone grafts, are often limited by complications including secondary donor site morbidity, prolonged rehabilitation periods, and high rates of graft resorption (reported to be as high as 30% in some studies). Specific complications of traditional bone grafting include infection at the donor site, failure of graft integration, and mechanical instability, leading to the need for repeated surgeries [2]. For instance, studies have shown that iliac crest bone grafting, one of the common methods, may lead to chronic pain and gait disturbances, contributing to overall morbidity. Additionally, bone grafts may not provide adequate support for prostheses, particularly in patients with extensive defects resulting from mucormycosis.

Addressing the complex anatomical and functional challenges during rehabilitation is essential, as patients frequently face issues such as insufficient maxillary bone, involvement of the pterygoid plates, and occasionally the zygomatic bone, adherence of the nasal and sinus mucosa to the palatal mucosa, fibrosed palatal tissue, loss of lip support, a diminished stress-bearing area, inadequate vertical guidance, and mandibular overclosure. While quad zygoma implants remain a valid treatment option, they may not be feasible for patients with zygomatic bone resected due to necrosis. Subperiosteal implants, which are positioned on the bone just beneath the periosteum, present an alternative solution with a lower risk of bone resorption.

In today's context, virtual planning and digital three-dimensional (3D) printing technology can produce patient-specific drilling guides or plates to facilitate the placement of zygomatic implants, thus promoting effective prosthodontic rehabilitation [3]. These digital approaches significantly improve accuracy and reduce surgical complications. For completely or partially edentulous patients who have undergone total or subtotal maxillary resection, an obturator prosthesis remains a convenient and efficient removable prosthetic option.

Mommaerts introduced a novel concept for an additive-manufactured subperiosteal jaw implant, utilizing advanced computer-aided design and manufacturing (CAD/CAM) technology [4]. This approach offers an alternative implant solution for patients with significant jawbone atrophy or extensive defects following resection. CAD/CAM enables the precise customization of subperiosteal implants, providing better adaptation to the individual anatomical structure of each patient. However, tissue conditions post-oncological resection and subsequent radiotherapy necessitate a tailored design, as inadequate bone may limit the support even for subperiosteal implants.

## Case presentation

**Figure 1:**
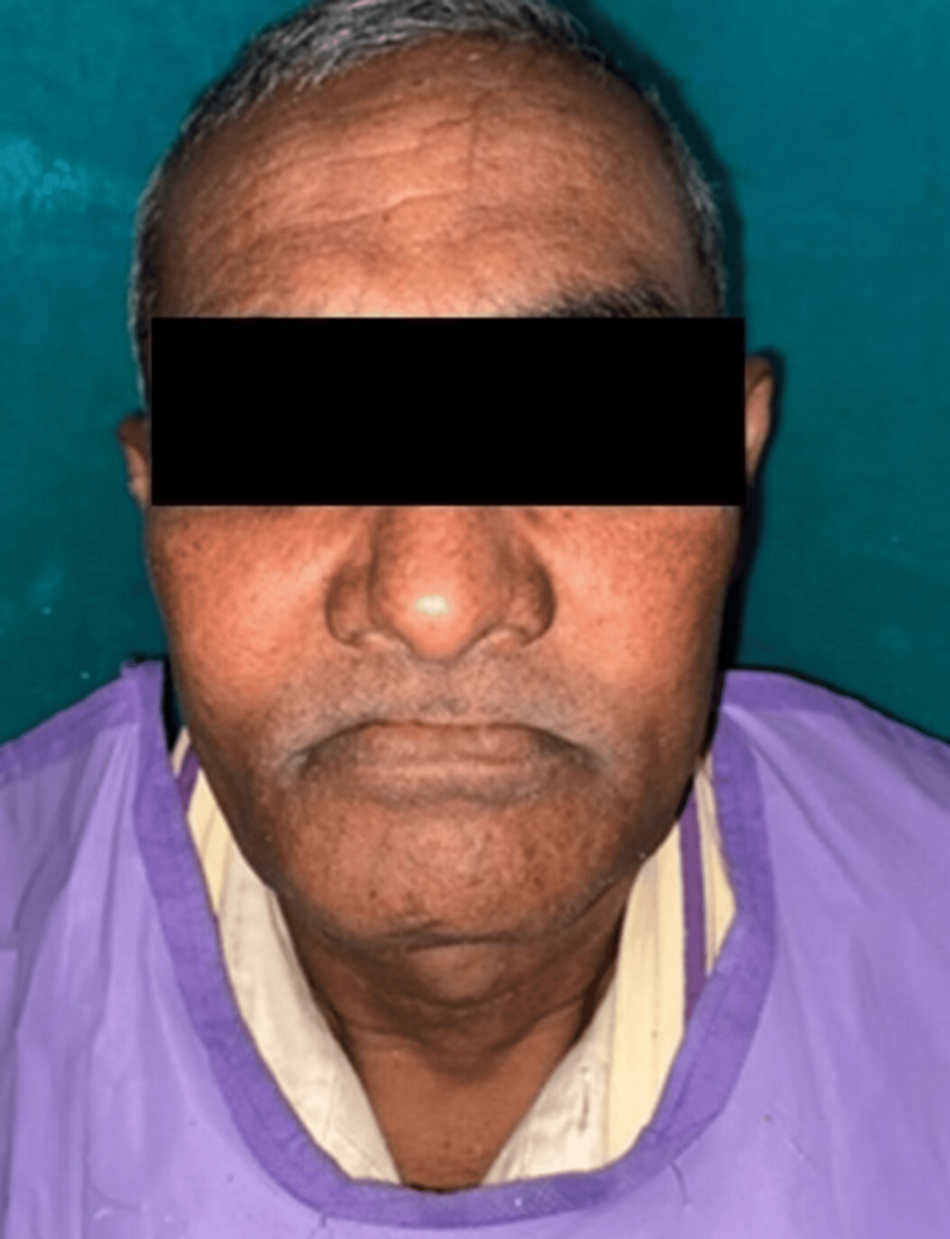
Pre-prosthetic extraoral photograph Extraoral photograph of the patient after resection of the inferior portion of the maxilla

A 52-year-old male patient presented to the Department of Prosthodontics and Crown and Bridge at Subharti Dental College and Hospital, with complaints of food regurgitation into the nasal cavity, difficulties with speech and mastication, and impaired aesthetics and phonetics, persisting for four months (Figure 1).

**Figure 2:**
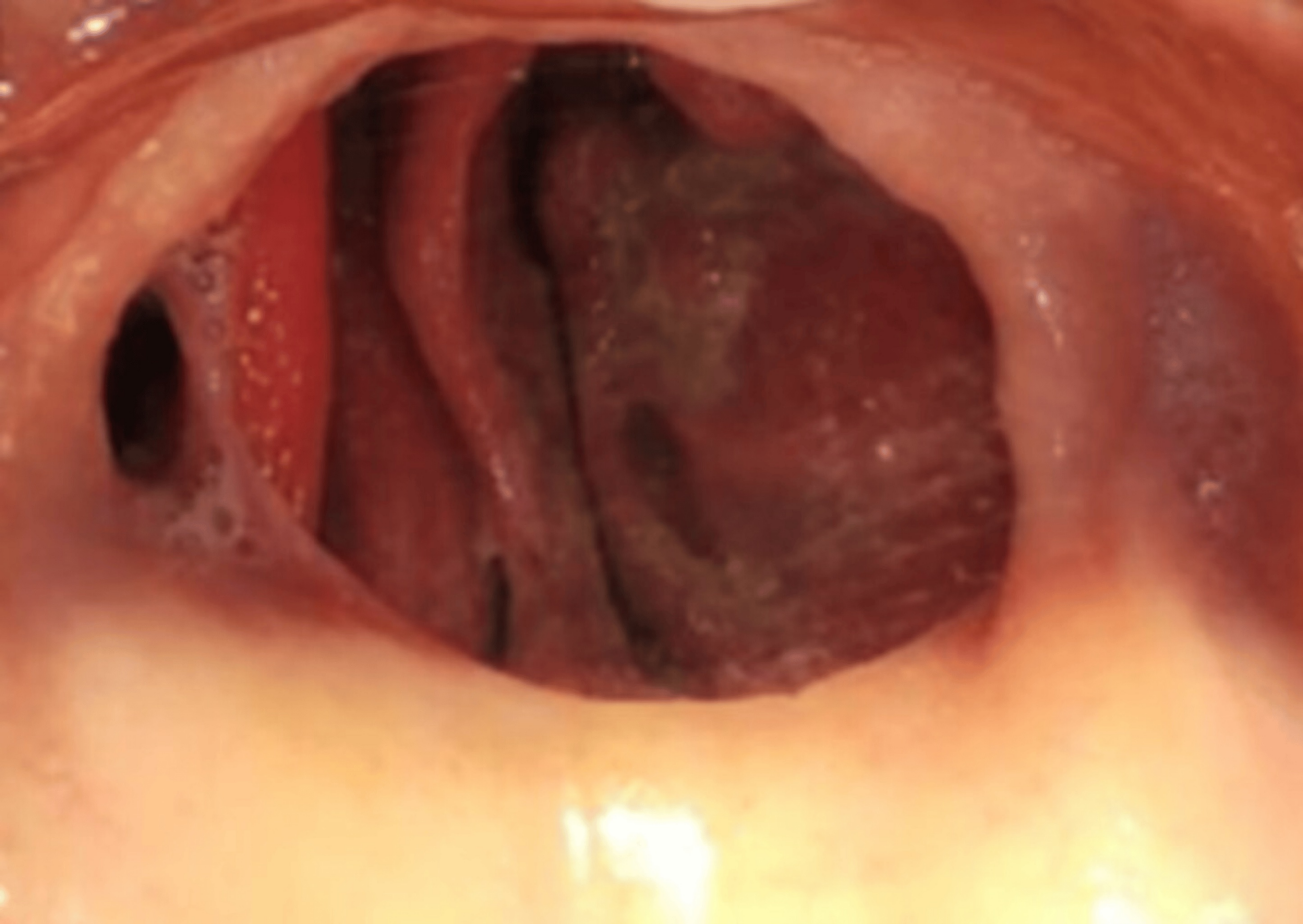
Pre-prosthetic intraoral photograph Intraoral photograph of the defect in the maxilla due to surgical resection

The patient had a significant medical history, including a diagnosis of COVID-19 and subsequent rhinocerebral mucormycosis, which affected the floor of the orbit, antrum, and hard palate. He underwent aggressive treatment for mucormycosis, which included antifungal therapy and surgical resection of the lower portion of the maxilla, resulting in an oro-antral communication. The disease was effectively controlled, and he had been clinically, radiographically, and pathologically disease-free for over six months following the maxillary resection (Figure 2).

However, the patient continued to experience functional impairments, including nasal regurgitation, compromised speech, and difficulties with mastication, significantly impacting his quality of life.

To restore his speech and swallowing functions, an obturator prosthesis was initially placed perioperatively. However, the patient experienced complications with the prosthesis, including frequent loosening, which negatively affected speech and swallowing, further reducing his quality of life. Traditional implant solutions, such as quad zygoma implants, were not viable due to inadequate bone support following the extensive resection, which included part of the zygomatic bone. Therefore, a customized subperiosteal zygoma implant was designed specifically for his unique anatomical needs.

**Figure 3:**
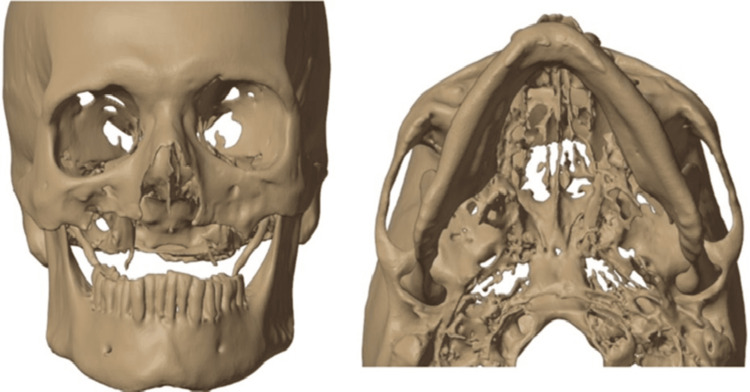
3D model of the skull 3D model of the skull obtained using CT 3D: three-dimensional; CT: computed tomography

First, the patient's post-surgical anatomy was visualized by segmenting pre- and post-maxillectomy computed tomography (CT) data. The study focused on extracting a 3D model of the patient's maxilla. The Digital Imaging and Communications in Medicine (DICOM) data from the CT scan was converted into a 3D model, and segmentation was performed using the DICOM software MIMICS 24.0 (Materialise NV, Leuven, Belgium). The resulting 3D model was then exported in Standard Tessellation Language (STL) format (Figure 3).

**Figure 4:**
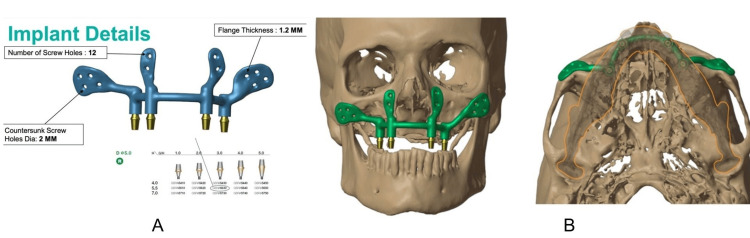
(A) Planning of the framework design. (B) Virtual framework in position (A) It shows the planning for the thickness of the framework and screw hole positions along with its diameter (B) It shows the virtual planning for the positioning of the framework on the 3D skull model 3D: three-dimensional

The virtual prosthesis model was aligned with the patient's anatomical 3D models to serve as the foundation for designing the patient-specific subperiosteal implant (psSPI). ​The intended placement of the prosthetic dental arch played a crucial role in determining the locations of the four attachments (Figure 4A, 4B).

**Figure 5:**
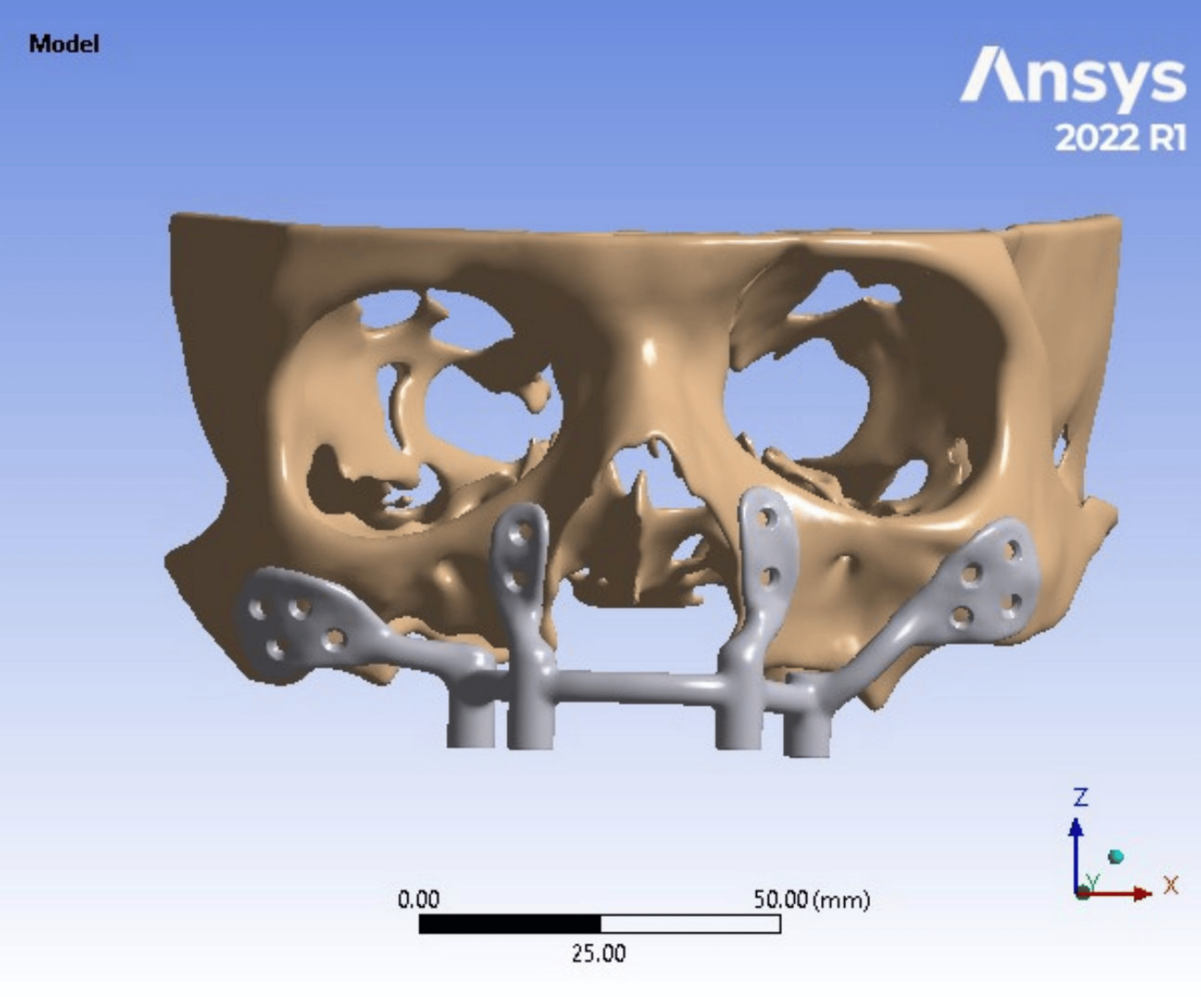
Finite element analysis Finite element analysis done for the evaluation of deformation under loading conditions

A U-shaped framework was designed on these attachments to support the obturator prosthesis. The psSPI was manufactured in medical-grade titanium alloy, i.e., Ti-6Al- 4V (Jajal Medical Services, Vadodara, Gujarat, India). Threads matching 2 mm locking screws were added to the screw holes. A flange thickness of 1.2 mm at the zygomatic area and the countersunk screw holes were 2 mm in diameter. An opening in the oral mucosa was provided for the stud attachments. A removable prosthesis was planned for the patient. Before the 3D printing of the framework using the direct metal laser sintering (DMLS) technique, a finite element analysis was done to determine the impact of loading the psSPI on the bone using the Ansys 2022 R1 software (Ansys, Inc., Canonsburg, Pennsylvania, United States) (Figure 5).

**Figure 6:**
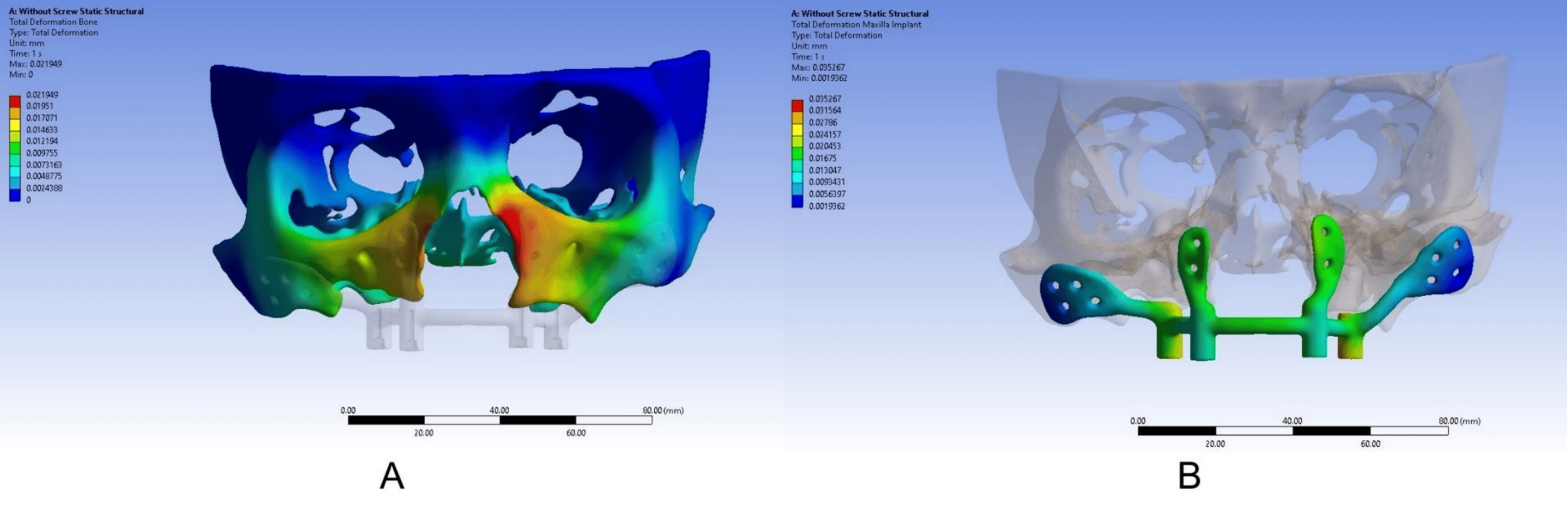
(A) Analysis of the deformation of the maxilla under 300 N loading condition. (B) Analysis of the deformation of the implant under 300 N loading condition (A) It shows the amount of deformation of the maxilla under 300 N loading (B) It shows the amount of deformation of the implant itself under 300 N loading

A load of 300 N, commonly cited in the literature as the average masticatory force in the molar region, was applied during the analysis [5] (Figure 6A, 6B).

**Figure 7:**
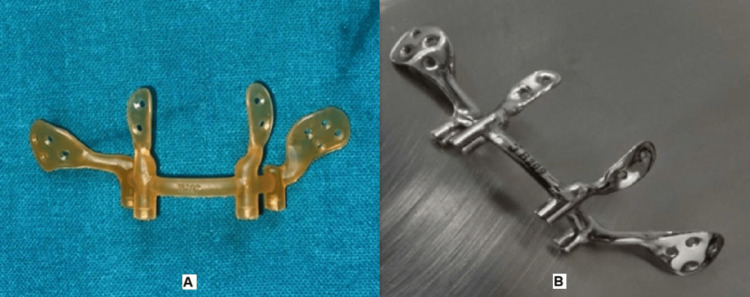
(A) 3D printed prototype model. (B) 3D printed framework fabricated by the DMLS technique 3D printed prototype model of the framework followed by its fabrication using the DMLS technique with medical-grade V titanium alloy 3D: three-dimensional; DMLS: direct metal laser sintering

The maximum stress on the maxilla remained within the allowable stress limit, indicating a low likelihood of mandibular failure under this load. A prototype model made of polylactic acid was used to conduct a mock surgery, during which screw fixation was performed (Figure 7A). Screw holes were designed to accommodate 2 mm locking screws in the 3D printed framework (Figure 7B).

The actual surgical procedure was planned to be conducted under general anesthesia, following a healing period after the maxillary resection. The timeline between the maxillary resection and the implant surgery was approximately six months, allowing for complete healing and ensuring that the patient was disease-free before moving forward with the prosthetic phase. This interval was critical for both the soft tissue and bone to recover adequately, facilitating a more predictable prosthetic outcome.

**Figure 8:**
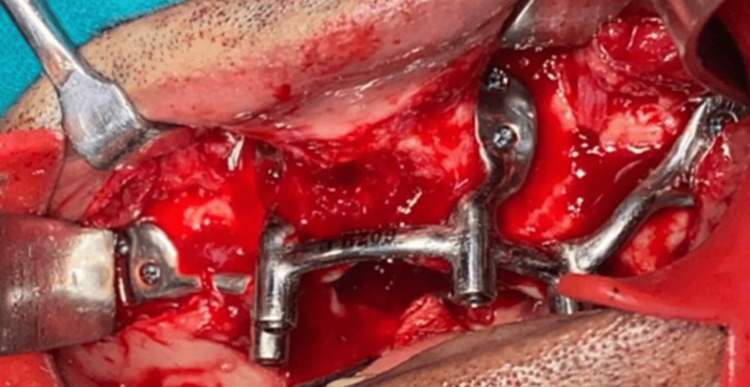
Surgical placement of the framework Surgical placement of the framework under general anesthesia

Under general anesthesia, a full-thickness flap was created to reveal the remaining zygomatic bone. Following this, the stability and proper alignment of the bone-supported implant were assessed. The holes for the locking screws were drilled. After careful alignment of the implant to the surgical site, it was screwed using Ortho Max screws (Ortho Max Manufacturing Co. Pvt. Ltd., Vadodara, Gujarat, India) (Figure 8).

**Figure 9:**
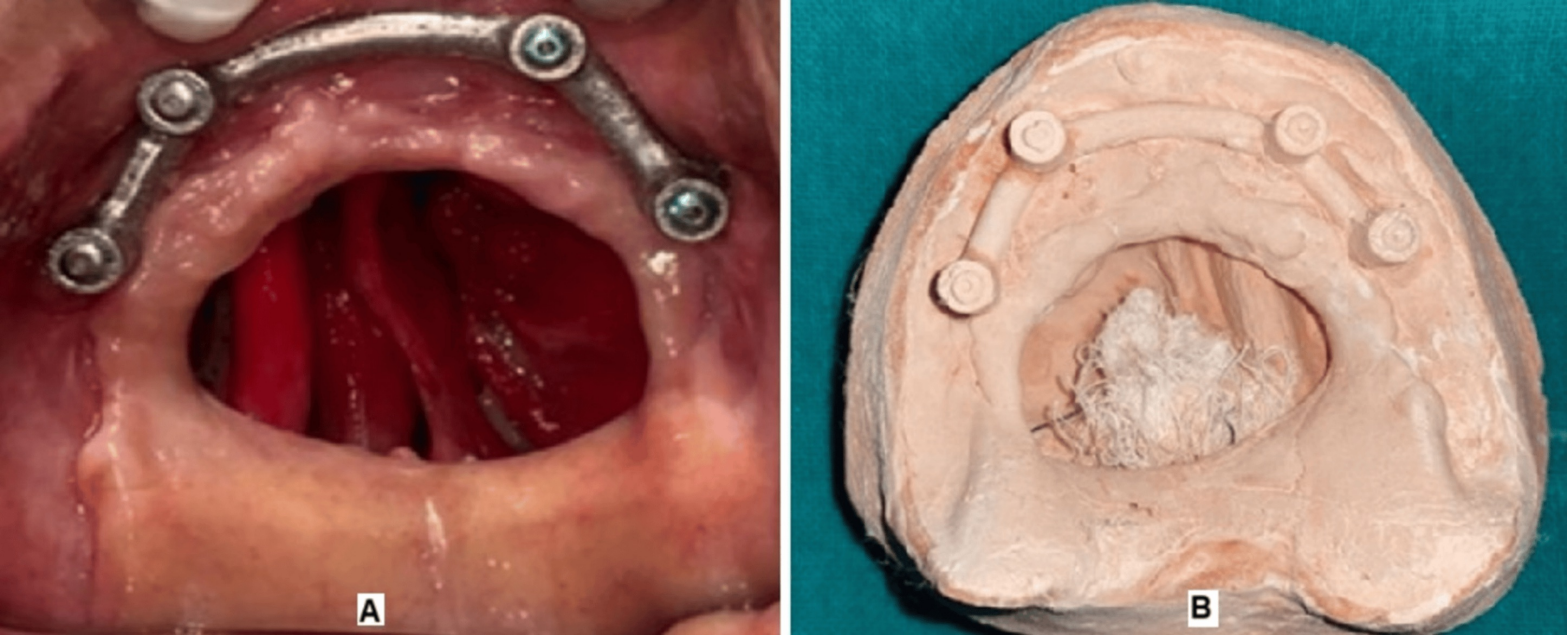
(A) Postoperative intraoral photograph. (B) Cast model (A) It shows the completely healed postoperative intraoral photograph (B) It shows the cast model of the maxilla which is to be rehabilitated

An obturator was planned two weeks after the surgical procedure post-initial soft tissue healing (Figure 9A). In this procedure, first, the undercuts were blocked with gauze, and a primary impression of the defect was made using irreversible hydrocolloid (Zhermack Tropicalgin Alginate, Zhermack SpA, Badia Polesine, Italy) and poured into type III dental stone (Kalabhai Kalstone, Kalabhai GmbH, Gelnhausen, Germany). A special tray was then fabricated, and the final impression was taken using medium-body elastomeric impression material, which was poured into type IV dental stone (Kalabhai Kalrock, Kalabhai GmbH, Gelnhausen, Germany) (Figure 9B).

**Figure 10:**
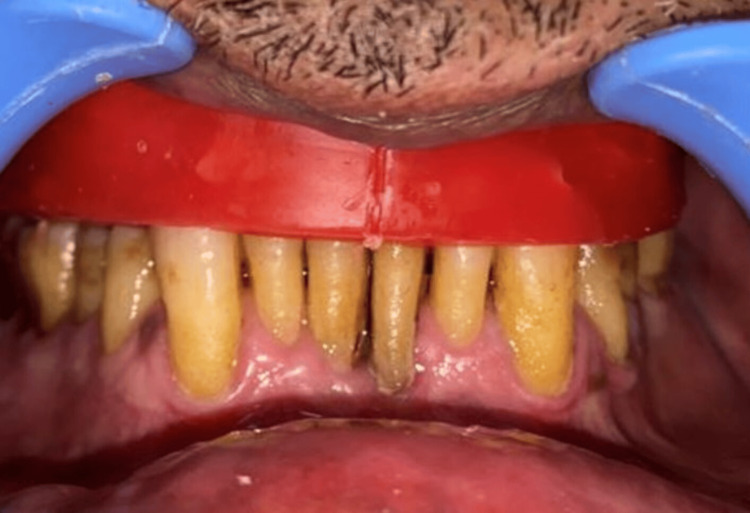
Jaw relation Jaw relation was recorded at the established vertical dimension

Jaw relation was recorded (Figure 10).

**Figure 11:**
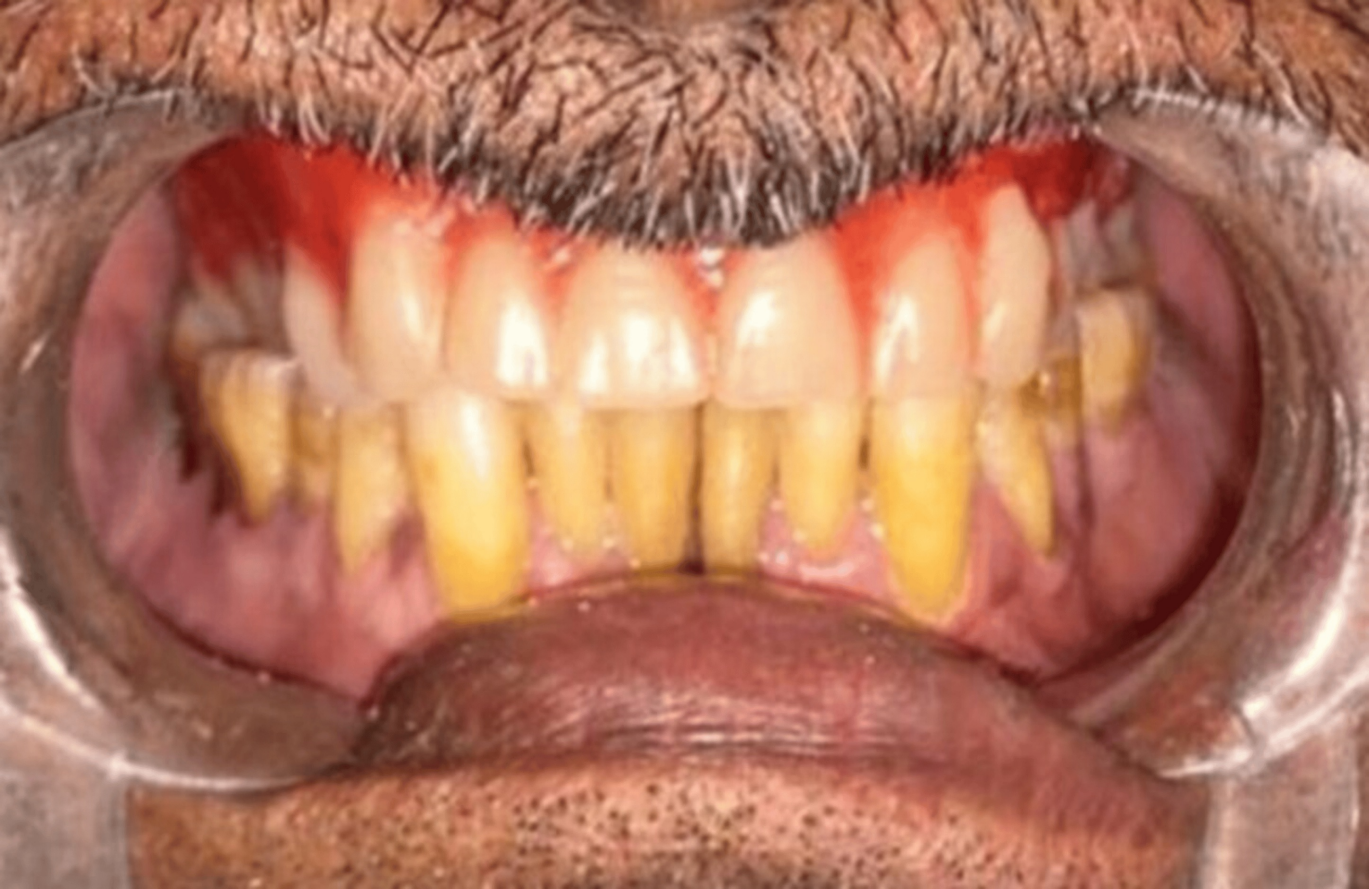
Try-in of the prosthesis Teeth arrangement done on the articulator followed by the try-in of the prosthesis in the patient's mouth

Teeth arrangement was done on an articulator and the try-in procedure was completed (Figure 11).

**Figure 12:**
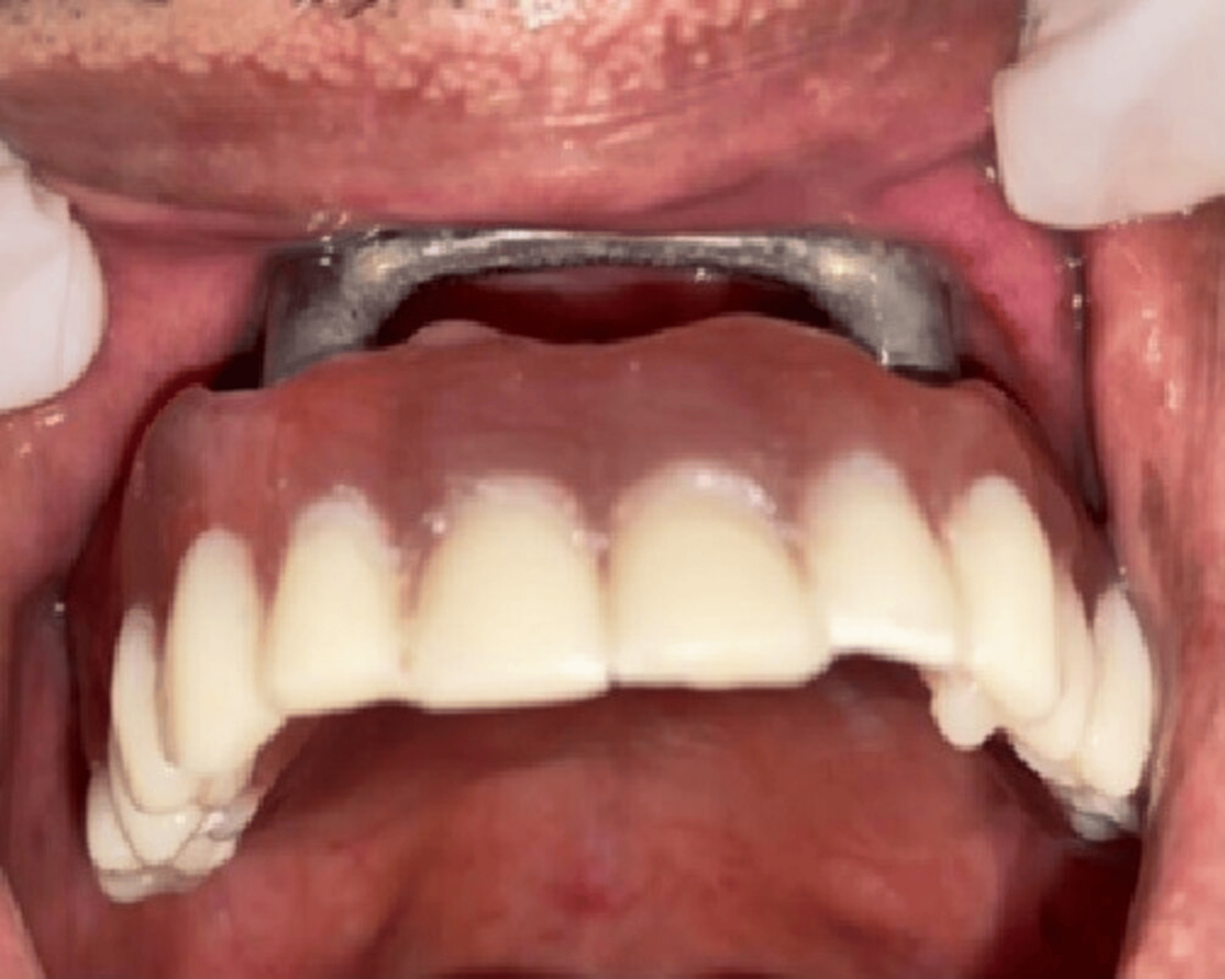
Definitive obturator: intraoral photograph Insertion of the attachment-retained obturator was done in the patient's mouth

The attachments were picked up using O-ring attachments (ABA Technologies, New Delhi, India) and the obturator was inserted (Figure 12). 

**Figure 13:**
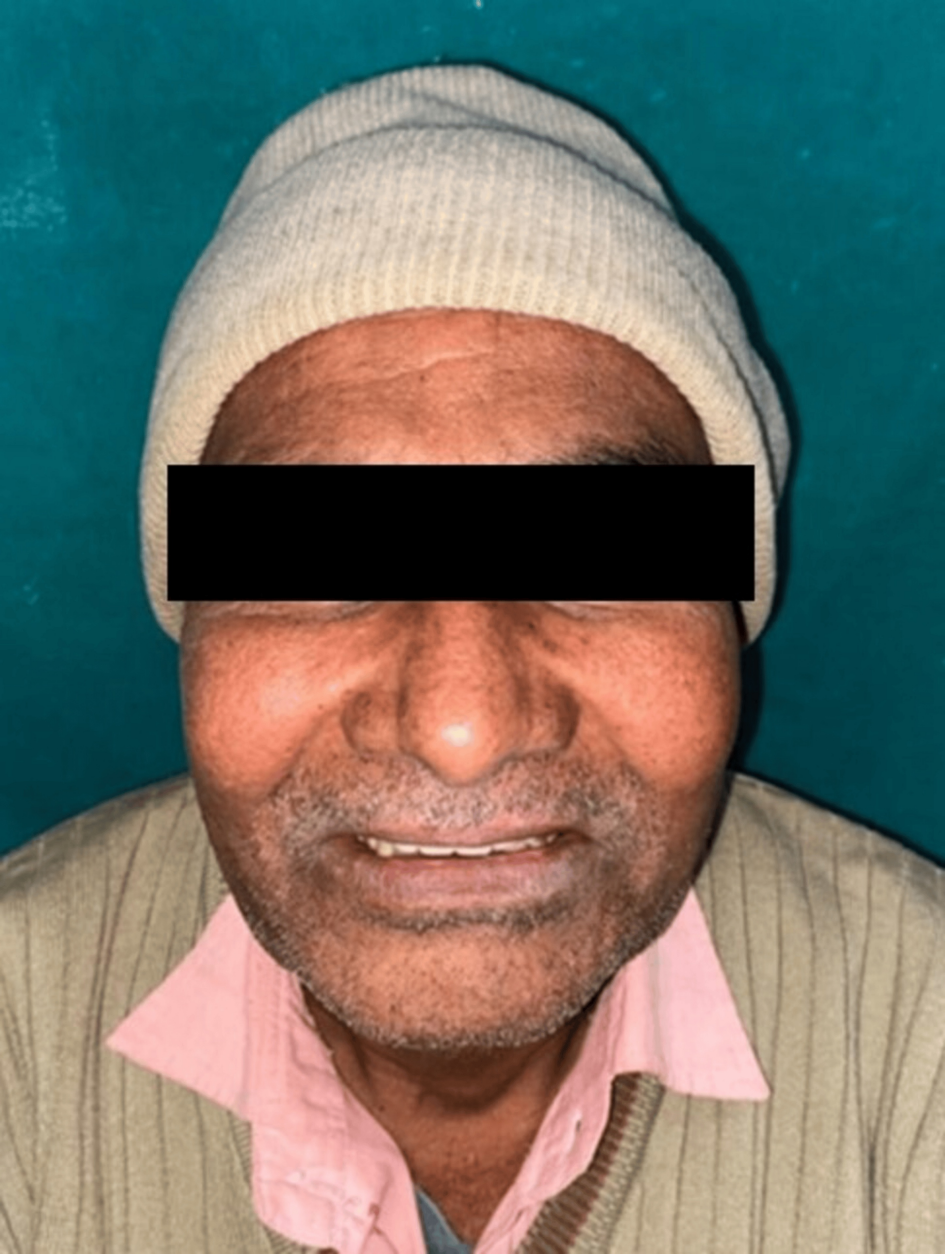
Post-insertion extraoral photograph Extraoral photograph of the patient wearing the obturator

During the three-month follow-up, the patient expressed satisfaction with the prosthesis, despite experiencing minor issues such as regurgitation, nasal leakage, and wear of the attachments. These issues were managed with minor adjustments and guidance on prosthesis maintenance. The patient reported feeling more confident and comfortable while wearing the prosthesis with significant improvements in speech, mastication, and overall functionality. Additionally, the enhanced facial aesthetics contributed positively to the patient's social interactions and psychological well-being, leading to an overall improved quality of life (Figure 13).

## Discussion

Post-COVID-19 mucormycosis results in significant anatomical deficits, substantial financial challenges, and lasting emotional impacts for individuals affected. The rehabilitation of such patients presents a considerable challenge for maxillofacial surgeons and prosthodontists, particularly due to the extensive size of the defects and the complexities of the anatomical structures involved [6]. ​A custom-designed prosthesis-driven patient-specific implant (PSI) offers a viable solution for addressing maxillectomy defects while minimizing morbidity and improving functional adaptability [7].​

CAD of the PSI enhances accuracy, compatibility with the defect, stability, predictability of results, and refinement of facial contours. In cases with severely compromised maxillary bone anatomy where traditional reconstruction methods are impractical, a PSI-retained prosthesis can effectively restore speech and oral function. A notable advantage of this approach is the removable obturator prosthesis, which facilitates thorough tissue examination and allows patients to independently maintain and clean both the prosthesis and the peri-implant tissues [8].

Titanium is recognized as a biocompatible and inert material that integrates well with the surrounding bone, which has made it the material of choice for internal maxillomandibular fixations and dental implants over recent decades [9].​ Its established biocompatibility has solidified its role in maxillofacial internal fixation and reconstruction. The use of titanium, as opposed to previously utilized chrome cobalt alloys, for subperiosteal implants enhances tissue compatibility and significantly decreases the chances of implant exposure and rejection [10]. Titanium has superior strength and lightweight properties, which are essential for long-term use in the oral cavity. Its excellent osseointegration properties also contribute to the stability and longevity of the prosthesis, reducing the risk of implant failure [11]. Although subperiosteal implants have previously lost popularity due to issues such as severe inflammation and inadequate fixation, the design implemented in this instance provided an effective alternative for a patient lacking viable treatment options, leading to improvements in oral function and overall oral health-related quality of life.

Due to the digitally engineered design of the implant, a functional prosthesis can be delivered in a single-stage surgical procedure. The implant features an integrated connection system that attaches to the resilient zygomatic bone, facilitating the immediate placement of the prosthesis onto the bar affixed to the implant [12]. The main advantage of employing PSIs is their ability to accurately restore 3D anatomy without the need for bone grafting procedures. This eliminates the complexities and time consumption associated with tissue transfer and reduces the risk of complications at the donor site [13]. Finite element analysis has shown that the psSPI can effectively endure occlusal loading. ​Therefore, it is advisable to use digitally planned psSPIs to support the closure of extensive maxillary defects in patients where direct or delayed restoration options are impractical.

## Conclusions

This case report highlighted the effective use of a digitally planned, patient-specific subperiosteal zygoma implant for maxillary rehabilitation following mucormycosis resection. Utilizing advanced CAD/CAM technology, a custom-fit implant was created, resulting in significant improvements in the patient's speech, mastication, and overall quality of life. The removable obturator prosthesis provided additional advantages, enabling easy maintenance and examination of the peri-implant tissues. This innovative approach offers a promising alternative for patients with extensive maxillary defects, presenting a viable solution when traditional reconstruction methods are not feasible. Looking forward, further research should focus on refining the design and fabrication processes of subperiosteal implants, including long-term clinical studies to assess their durability and success in diverse patient populations. Additionally, exploring the integration of other digital technologies, such as augmented reality for surgical planning, could enhance precision and outcomes in similar complex cases.
